# Aptamers: Biomedical Interest and Applications

**DOI:** 10.3390/ph10010032

**Published:** 2017-03-16

**Authors:** Cristina Romero-López, Alfredo Berzal-Herranz

**Affiliations:** Instituto de Parasitología y Biomedicina “López-Neyra”, (IPBLN-CSIC), 18016 Armilla, Granada, Spain

Aptamers are short DNA or RNA oligonucleotides specialized in the specific and efficient binding to a target molecule. They are obtained by in vitro selection or evolution processes. It was in 1990 that two independent research groups described the bases of a new in vitro technology for the identification of RNA molecules able to specifically bind to a target [1,2]. Tuerk and Gold established the principals of the in vitro selection process that was named SELEX (Systematic Evolution of Ligands by Exponential enrichment), which is based on iterative cycles of binding, partitioning, and amplification of oligonucleotides from a pool of variant sequences [2]. Ellington and Szostak coined the term aptamer to define the selected molecules by the application of this method [1]. To date, numerous reports have described the isolation of aptamers directed against a great variety of targets covering a wide diversity of molecules varying in nature, size, and complexity ranging from ions to whole cells, including small molecules (e.g., aminoacids, nucleotides, antibiotics), peptides, proteins, nucleic acids, and viruses, among others (for example, see [3,4,5,6]). Modifications and optimization of the SELEX procedure aimed to get newly modified aptamers has also attracted much interest (examples can be found in [7,8]). These advances along with the parallel progresses in the nucleic acids chemistry and cellular delivery fields have allowed for the rise of a new hope in developing aptamers as efficient molecular tools for diagnostics and therapeutics (for recent comprehensive reviews, see [9,10,11]).

Proof of the great potential and interest of aptamers and their applications has been the success of a recent meeting entitled “Aptamers,” which took place in the charming French city of Bordeaux last June, as well as the announcement of a second meeting to occur in September 2017. The latest achievements in the field and new technical developments applied to the selection, sequence analysis, and/or aptamer applications were presented. Different aspects of the scientific content, together with other details of the Bordeaux meeting, have been brilliantly summarized by members of the Scientific Committee in the form of a Meeting Report that has been included in this special issue [12].

This issue also provides four review articles that summarize the state of the art of different aspects of aptamer technology. The first two articles review the latest advancements in the potential application of aptamers to solve two problems of great biosanitary importance: cancer immunotherapy [13] and viral infections [14]. Pastor’s review provides a detailed analysis of reported aptamer assays with antagonist activity of the immune checkpoint in cancer, as well as those focusing on aptamers as new enhancer compounds of the immune system. The author also accounts for the use of aptamers in clinical cancer immunotherapy in the near future [13]. The review of González et al. [14] provides evidence for the potential use of aptamers in the development of diagnostic platforms for viral infections and antiviral agents. This review summarizes examples of the successful use of aptamers targeting both viral proteins and specific domains of the viral genome. They also predict a promising future in aptamer technology for the treatment and diagnosis of viral diseases [14].

The third article provides an update on the application of aptamers as drug delivery agents [15]. The authors very nicely address other interesting uses of aptamer technology. Instead of pursuing the direct targeting of a non-desired molecule in the search of therapeutic activity, researchers can select aptamers against cellular receptors to promote the delivery of a specific drug in cells overproducing the aptamer target-receptor. This represents another interesting use for these molecules and thus significantly expands the portfolio of potential biomedical applications of aptamers. The authors conclude that pursuing the study of the still not well known aspects of the aptamer-mediated internalization or drug release, as well as a rigorous toxicological study, should certainly lead to the development of an efficient aptamer-mediated drug delivery therapy [15].

The great development of high-throughput sequencing strategies has been of significant help to aptamer technology. The application of these strategies to the selected pools of molecules provides an excellent tool for the faster and safer identification of the best aptamers against a particular target. It also ensures a more complete knowledge of population complexity after each round of selection, allowing for conclusions about the effectiveness of the selection procedure. The application of high-throughput sequencing techniques for aptamer characterization has been reviewed in an article by F. Ducongé’s group [16]. The authors think that, beyond the existing drawbacks—mainly the high cost of applying these sequencing techniques—their application provides advantages that significantly improve or optimize the procedure of aptamer identification. Pursuing the development of new apparatus and of new software that could provide high-throughput for both sequencing and binding analysis would be of great help for the desired routine isolation of aptamers for clinical use [16].

Finally, this issue also includes a research article by Gijs et al. [17], wherein they describe the application of a SELEX-based procedure to the isolation of DNA aptamers specific to the HER2 receptor, a protein that is commonly exposed on the surface of different tumor cells. This manuscript shows evidence that the cellular internalization of a selected aptamer via HER2 leads to the inhibition of cancer cell growth and affects cellular viability [17]. These results allow the authors to propose the potential of these DNA aptamers as candidates for the development of novel HER2-positive cancer diagnosis systems and for the design of therapeutic agents.

This special issue encompasses concrete examples of applications of aptamers to biomedical challenges, like diagnoses and therapeutics of cancer and viral infections. They can be extrapolated to other disease models and even to biotechnological problems. The issue also provides an example of how aptamers can be used as tools for the development of novel technologies, as well as for the application of last-generation strategies of sequence analysis for the optimization of aptamer technology. Finally, the inclusion of the Bordeaux meeting report allows this issue to cover all of the most significant aspects of the aptamer field, providing references to all of the latest advances mentioned herein.

## References

[1] Ellington A.D., Szostak J.W. (1990). In vitro selection of RNA molecules that bind specific ligands. Nature.

[2] Tuerk C., Gold L. (1990). Systematic evolution of ligands by exponential enrichment: RNA ligands to bacteriophage t4 DNA polymerase. Science.

[3] Wilson D.S., Szostak J.W. (1999). In vitro selection of functional nucleic acids. Annu. Rev. Biochem..

[4] Rajendran M., Ellington A.D. (2008). Selection of fluorescent aptamer beacons that light up in the presence of zinc. Anal. Bioanal. Chem..

[5] Raddatz M.S., Dolf A., Endl E., Knolle P., Famulok M., Mayer G. (2008). Enrichment of cell-targeting and population-specific aptamers by fluorescence-activated cell sorting. Angew. Chem. Int. Ed. Engl..

[6] Kumar P.K. (2016). Monitoring intact viruses using aptamers. Biosensors (Basel).

[7] Marton S., Reyes-Darias J.A., Sanchez-Luque F.J., Romero-Lopez C., Berzal-Herranz A. (2010). In vitro and ex vivo selection procedures for identifying potentially therapeutic DNA and RNA molecules. Molecules.

[8] Lipi F., Chen S., Chakravarthy M., Rakesh S., Veedu R.N. (2016). In vitro evolution of chemically-modified nucleic acid aptamers: Pros and cons, and comprehensive selection strategies. RNA Biol..

[9] Vorobyeva M., Vorobjev P., Venyaminova A. (2016). Multivalent aptamers: Versatile tools for diagnostic and therapeutic applications. Molecules.

[10] Nimjee S.M., White R.R., Becker R.C., Sullenger B.A. (2017). Aptamers as therapeutics. Annu. Rev. Pharmacol. Toxicol..

[11] Zhou J., Rossi J. (2017). Aptamers as targeted therapeutics: Current potential and challenges. Nat. Rev. Drug Discov..

[12] Toulme J.J., Giangrande P.H., Mayer G., Suess B., Duconge F., Sullenger B., de Franciscis V., Darfeuille F., Peyrin E. (2017). Aptamers in bordeaux, 24–25 June 2016. Pharmaceuticals (Basel).

[13] Pastor F. (2016). Aptamers: A new technological platform in cancer immunotherapy. Pharmaceuticals (Basel).

[14] Gonzalez V.M., Martin M.E., Fernandez G., Garcia-Sacristan A. (2016). Use of aptamers as diagnostics tools and antiviral agents for human viruses. Pharmaceuticals (Basel).

[15] Catuogno S., Esposito C.L., de Franciscis V. (2016). Aptamer-mediated targeted delivery of therapeutics: An update. Pharmaceuticals (Basel).

[16] Nguyen Quang N., Perret G., Duconge F. (2016). Applications of high-throughput sequencing for in vitro selection and characterization of aptamers. Pharmaceuticals (Basel).

[17] Gijs M., Penner G., Blackler G.B., Impens N.R., Baatout S., Luxen A., Aerts A.M. (2016). Improved aptamers for the diagnosis and potential treatment of her2-positive cancer. Pharmaceuticals (Basel).

